# Antimalarial Activity of *Cordia africana* (Lam.) (Boraginaceae) Leaf Extracts and Solvent Fractions in *Plasmodium berghei*-Infected Mice

**DOI:** 10.1155/2019/8324596

**Published:** 2019-08-18

**Authors:** Dawit Zewdu Wondafrash, Dayananda Bhoumik, Birhanetensay Masresha Altaye, Helen Bitew Tareke, Brhane Teklebrhan Assefa

**Affiliations:** ^1^Department of Pharmacology and Toxicology, School of Pharmacy, Mekelle University, Mekelle, Ethiopia; ^2^College of Medicine, Debre Berhan University, Debre Berhan, Ethiopia; ^3^Department of Pharmacognosy, School of Pharmacy, Mekelle University, Mekelle, Ethiopia

## Abstract

**Background:**

Malaria remains a major worldwide public health problem leading to death of millions of people. Spread and emergence of antimalarial drug resistance are the major challenge in malaria control. Medicinal plants are the key source of new effective antimalarial agents. *Cordia africana* (Lam.) is widely used for traditional management of malaria by local people in different parts of Ethiopia. The present study aimed to evaluate *in vivo* antimalarial effects of leaf extracts and solvent fractions of *Cordia africana* on *Plasmodium berghei*-infected mice.

**Methods:**

The leaf extracts were prepared and tested for oral acute toxicity according to the OECD guideline. *In vivo* antimalarial effects of various doses of *C. africana* extracts and solvent fractions were determined using the four-day suppression test (both crude and fractions), as well as curative and chemoprophylactic tests (crude extracts).

**Results:**

The acute toxicity test of the plant extract revealed that the medium lethal dose is higher than 2000 mg/kg. The crude extract of the plant exhibited significant parasitemia suppression in the four-day suppression (51.19%), curative (57.14%), and prophylactic (46.48%) tests at 600 mg/kg. The *n*-butanol fraction exhibited the highest chemosuppression (55.62%) at 400 mg/kg, followed by the chloroform fraction (45.04%) at the same dose.

**Conclusion:**

Our findings indicated that both the crude leaf extracts and fractions of *C. africana* possess antimalarial effects, supporting the traditional claim of the plant.

## 1. Introduction

Malaria is a life threatening infectious disease caused by unicellular eukaryotic protozoan parasites belonging to the genus *Plasmodium*, which is a member of the phylum Apicomplexa [1, 2]. The disease remains a major cause of morbidity and mortality with more than 3.2 billion people affected worldwide [3]. Besides, it takes the lives of people in the tropical and subtropical part of the world each year, especially in the Sub-Saharan Africa, and most of the victims are children below five years of age and pregnant women [4]. The main cause of the worsening malaria situation in recent years has been the spread of drug-resistant parasites. This has led to escalating malaria-associated death [5].

Popular knowledge of medicinal plants used by humans is based on thousands of years of experience. Developing countries possess a wide range of potentially useful medicinal plants, which are more extensive indeed than available in many other parts of the world [6]. Nowadays, the World Health Organization (WHO) estimates that eight percent of the population of Asian and African countries uses herbal medicine for various aspects, primarily for health care [7]. In Ethiopia, about eight percent of the population is still dependent on traditional medicine, which predominantly encompasses the use of plants. The analysis of those medicines employed for the management of malaria represents a potential for investigation of lead molecules and development of antimalarial drugs [8].

*Cordia* is a pantropical genus of flowering plants that belongs to Boraginaceae family. The plant family comprises about 100 genera and more than 2000 species [9], from which *Cordia africana* (Lam.) is one of the common forest tree species in the family. It is commonly named as East African *Cordia* or large-leafed *Cordia* or Sudan teak in English, which is one of the main sources of traditional medicines serving to treat different diseases of human beings [9, 10]. Its fruits have a sweet and edible pulp with higher nutritional value [11, 12], and it was found to be a very good source of total phenol antioxidants [13].

The ethnomedicinal use of *C*. *africana* in treatment of various diseases has been validated in several experimental studies. Ethanol extracts of the plant exhibited *in vivo* anti-inflammatory [14] and antioxidant [10, 14, 15] and antinociceptive activities [16]. Another study on the leaf and stem bark extracts of *C. Africana* also showed that the plant possesses anti-inflammatory, antibacterial, and antioxidant activities [17].

Ethnopharmacological surveys conducted in different areas of Ethiopia showed the extensive use of *C. africana* against malaria [18–20]. In Jimma Zone, dried powder of the leaf is used to treat malaria and its related symptoms by traditional healers [21]. The extracts of *C. africana* have been reported to exhibit *in vitro* antiplasmodial activity against strains of *P. falciparum* [22].

Several studies reported that phytochemical analysis of the extracts of *C. africana* is a rich source of essential secondary metabolites [15, 23]. The present study was undertaken to investigate *in vivo* antimalarial activity of crude extracts and solvent fractions of *C. africana* leaves in chloroquine-sensitive *Plasmodium berghei*-infected mice.

## 2. Methods

### 2.1. Chemicals, Reagents, and Drugs

The following chemicals, reagents, and drugs were used in the present study: *n*-butanol (Fresenius Kabi®, India), absolute methanol (Loba chemie®, India), chloroform (Carlo Erba®, France), distilled water (Ethiopian pharmaceutical manufacturing (EPHARM), Ethiopia), normal saline (Addis Pharmaceutical Factory (APF), Ethiopia), Geimsa stain (BDH laboratory supplies Poole Gurr®, England), microscope immersion oil (Neolab Life Science Co., India), trisodium citrate (BDH Chemicals Ltd, England), and active chloroquine phosphate (APF, Ethiopia). All chemicals were of analytical grade and procured from certified suppliers.

### 2.2. Collection and Preparation of Plant Material

Fresh leaves of *C. africana* were collected from Chalacot, Tabia, Enderta, and Woreda which is located 17 kilometers South of Mekelle city and about 800 kilometers away from Addis Ababa on November 26, 2017. The plant was identified and authenticated by a taxonomist, and voucher specimens of the plant material were deposited at the National Herbarium, Department of Biology, Addis Ababa University, Ethiopia, for future reference with the voucher number designated as DZ001/2017.

The collected leaves were washed thoroughly with running tap water and air-dried at room temperature under shade. Then, the dried leaves of the plant material were chopped to smaller pieces and were ground into coarse powder using an electrical grinding mill. Then, the powdered leaves were kept in a tightly closed container until extraction.

### 2.3. Preparation of Crude Extract and Solvent Fractions

A total of 1200 gm of dried leaves was extracted by maceration (400 gm of coarsely powdered leaves was soaked in 2400 ml of 80% methanol). After 72 hours, the extract was first filtered using muslin cloth from the marc and then further filtered by the Whatman filter paper (no.1). Then, the filtrates from each extraction were combined together and then dried on a drying oven at a temperature of 40°C. Eventually, the dried extract was stored in amber glass in a refrigerator at 4°C for future use [24, 25].

A portion of the extract of *C. africana* (60 gm) was further fractionated with liquid-liquid extraction. Sequential solvent partitioning of the 80% methanol extract was conducted using a separatory funnel with equal volumes of chloroform and *n*-butanol, and the remaining solution was considered as an aqueous fraction. The three fractions were collected, dried, and kept in the same condition as the crude extract until use [26, 27].

### 2.4. Acute Toxicity Study

The acute oral toxicity test was conducted based on the Organization for Economic Cooperation and Development (OECD) guideline number 425 [28]. Accordingly, healthy, nulliparous, and nonpregnant five female Swiss albino mice aged 6–8 weeks were used. All mice were fasted for three hours before and an hour after the administration of the limited dose of the plant extract. A single female mouse was given 2000 mg/kg of the extract as a single dose by oral gavage. Since no death was observed in the first 24 hours, another 4 female mice were given the same dose. Following administration of the dose, the animals were closely observed, for any indication of gross physical and behavioral changes, in the first 4 hours and occasionally in a day for a total of 14 days.

### 2.5. Preparations of Experimental Animals and Rodent Parasites

Healthy adult male Swiss albino mice (weighing 25–35 gm and aged 6–8 weeks) were bred in animal house and maintained at the laboratory of the Department of Pharmacology and Toxicology, School of Pharmacy, Mekelle University. All animals were housed in an air-conditioned room of a 12-hour light/12-dark cycle. The mice were fed with standard pellet diet and water *ad libitum* throughout the experimental period.

The mice were acclimatized for a week to the laboratory environment prior to use. All the experiments were conducted in accordance with the internationally accepted laboratory animal use, care, and guideline [29]. The experiment was approved by the Health Research Ethics Review Committee (HRERC), College of Health Sciences, Mekelle University, with the protocol number ERC 1214/2018. Animals were humanely killed under anesthesia after completion of the follow-up period of each experiment.

The chloroquine-sensitive strain *Plasmodium berghei* (ANKA) was obtained from the Ethiopian Public Health Institute (EPHI), Addis Ababa University, and was maintained by a continuous serial intraperitoneal transfer of blood from infected to noninfected mice on a weekly basis.

### 2.6. Grouping and Dosing of Animals

**Figure 1:**
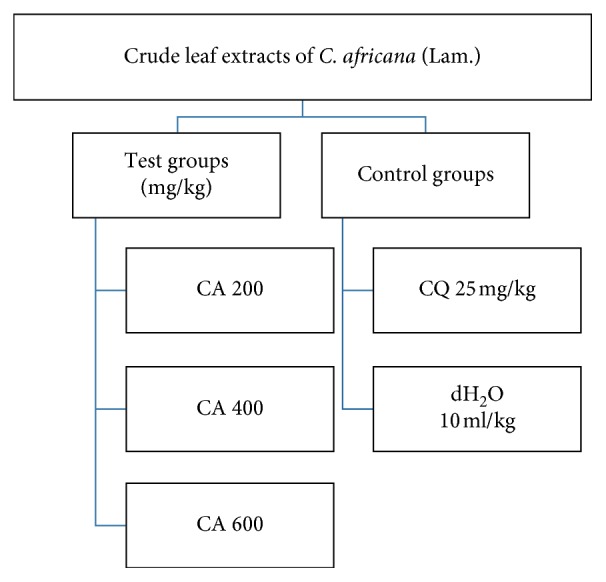
Grouping and dosing of crude leaf extracts of *C. africana* (Lam.). CQ, chloroquine; CA, *cordia africana*; dH_2_O, distilled water.

**Figure 2:**
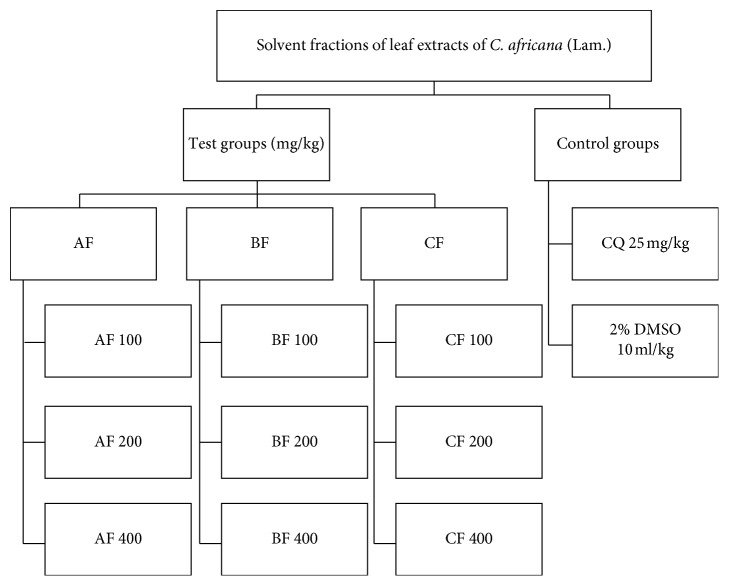
Grouping and dosing of the solvent fractions of the leaf extracts of *C. africana* (Lam.). AF, aqueous fraction; BF, *n*-butanol fraction; CF, chloroform fraction; CQ, chloroquine; DMSO, dimethyl sulfoxide.

Three antimalarial study models (four-day suppression, curative and prophylactic) were employed in the current study. As displayed in Figures 1 and 2, in each model, five groups of mice were used. For the solvent-fraction four-day suppression model, eleven groups of mice were used. The mice were randomly divided into five groups (negative control, positive control, and three test groups) comprising six animals per group. In all the experimental models, group I mice were administered with distilled water (dH_2_O) for crude extracts and 2% dimethyl sulfoxide (DMSO) for the solvent fractions and served as a negative control. Group V mice served as a positive control (treated with active chloroquine phosphate (CQ), 25 mg/kg). The test groups, groups II–IV, were treated with 200 mg/kg, 400 mg/kg, and 600 mg/kg for the crude extracts and 100 mg/kg, 200 mg/kg, and 400 mg/kg doses for the solvent fractions, respectively. Each dose was dissolved by dH_2_O/2% DMSO and administered through oral gavage.

### 2.7. Inoculation of Parasite

A donor mouse infected by chloroquine-sensitive *P. berghei* strain with a rising parasitemia 20–30% was used in all the tests. The mice were sacrificed and blood was collected in a Petri-dish with an anticoagulant (0.5% trisodium citrate) by severing the jugular vein. The blood was then diluted with normal saline (0.9%) based on parasitaemia level of the donor mice and the red blood cell count of normal mice, in such a way that 1 mL blood contains 5 × 10^7^ infected red blood cells. Each mouse was then inoculated with 0.2 mL of the diluted blood containing about 1 × 10^7^*P. berghei*-infected erythrocytes via the intraperitoneal route [24, 30].

### 2.8. *In Vivo* Antimalarial Activity Screening

#### 2.8.1. Test on Early Malaria Infection (Four-Day Suppression Test)

This test has been used to evaluate the schizontocidal effect of the crude leaf extracts and solvent fractions of *C. africana*. Five groups of male Swiss albino mice were inoculated with the parasite on day 0. The mice were then treated according to the grouping and dosing mentioned above. All the treatments were given through oral route by using oral gavage. Treatment started 3 hours after infection on day 0 and then continued daily for four consecutive days (i.e., from day 0 to day 3). On day 4 of the experiment, blood was collected from the tail of each mouse and thin smear was prepared on two microscope slides to determine parasitemia level. Body weight of each mouse was measured prior to parasite infection (day 0) and at the end of treatment (day 4). Rectal temperature was measured daily starting from day 0 till day 4. Furthermore, the mice were also followed for 30 days in order to record their survival time [31].

#### 2.8.2. Test on Established Malaria Infection (Curative or Rane's Test)

The chemotherapeutic activity of the crude extract of *C. africana* was carried out on established infection according to the method described by [32]. Thirty male mice were inoculated intraperitoneally with the standard inoculum of 1 × 10^7^*P. berghei*-infected erythrocytes on the first day (day 0). After 72 hours after infection, the mice were randomly divided into five groups and treated as described in the grouping and dosing section. Treatment was continued once daily for 7 days. The Giemsa-stained thin blood film was prepared from the tail of each mouse starting from day 3 and continued daily throughout the experiment to monitor parasitaemia level. Body weight was measured three hours before starting treatment (day 3) and at the end of treatment (day 7). Rectal temperature was measured daily starting just before treatment initiation (day 3) up to day 7 to see the effect of the extracts on body temperature. Thereafter, the mice were followed for 30 days, and their survival time was recorded.

#### 2.8.3. Test on Chemoprophylactic Activity (Repository Test)

Prophylactic potential of the crude leaf extract was evaluated based on residual infection procedure described by [33]. Adult male mice were weighed and randomly assigned into five groups. Mice were treated daily for four consecutive days as described under grouping and dosing section. Twenty-four hours after the last dose of treatment (day 0), the mice were inoculated intraperitoneally with 0.2 milliliters of infected blood containing 1 × 10^7^ inocula of *P. berghei*. After 72 hours (day 3), blood smears were made from the tail snip of each mouse and parasitemia level was determined. Body weight and rectal temperature were measured before inoculating parasite (day 0) and at the end of the experiment (day 3). Each mouse was then followed for one month to measure mean survival time.

### 2.9. Parasitaemia Measurement

Parasitaemia level was determined for all thin smears of blood films made from the tail of each mouse in all the three models. Two slides for each mouse were examined under a light microscope with a little drop of oil immersion objective of 100x magnification power, and then the number of parasitized erythrocytes in random fields of the microscope was counted. Four different fields were randomly selected on each of the slides and were used to calculate percent parasitaemia and percent suppression using the following formula, respectively [24, 25]:

(1) $$\begin{array}{c} \text{ \% parasitemia}=\;\frac{\text{number of parasitized red blood cells }}{\text{total number of red blood cell count }}\times \;100,\\ \text{\% suppression}=\left[\frac{A\;–\;B}{A\;}\right]\times 100, \end{array}$$

where *A* is the mean percent parasitaemia of the mice taken as negative controls and *B* is the mean percent parasitaemia in treatment groups.

### 2.10. Monitoring of Mean Survival Time

Mortality was monitored daily for 30 days (D0–D29), and the number of days from the time of inoculation of the parasite up to death was recorded for each mouse in all the models. The mean survival time (MST) for each group was calculated as follows [34]:

(2) $$\begin{array}{c} \text{MST}=\;\frac{\text{sum of the survival time of mice in a group }\left(\text{days}\right)}{\text{total of number of mice in that group}}. \end{array}$$

### 2.11. Determination of Body Weight and Temperature Change

The percent changes of mean values in body weight and rectal temperature that occur before and after treatment were calculated [35].

### 2.12. Statistical Analysis

The results of the study were expressed as the mean ± standard error of mean (SEM). Comparisons were made between the negative control, positive control, and treatment groups of various doses using ANOVA followed by Tukey's post hoc test with SPSS version 22.0. All data were analyzed at a 95% confidence interval. A *p* value less than 0.05 was considered statistically significant.

### 2.13. Ethical Approval

Ethical clearance was obtained from the Health Research Ethics Review Committee (HRERC) of the College of Health Sciences, Mekelle University, with protocol number ERC 1214/2018 on January 04, 2018 before the actual experimental activities commenced.

## 3. Results

### 3.1. Extraction Yields of Plant Material

The percent yield of the 80% methanol crude extract of the pulverized leaf (1200 gm) of *C. africana* was found to be 12.06% (w/w) with an actual yield of 144.72 gm. The extract was dark green colour and hygroscopic solid matter at room temperature. The fine powder was formed after drying in an oven, and then homogenization was done using mortar and pestle. Besides, the yield of each solvent fraction was also determined for the 60 gm of the crude extract. Accordingly, the highest percent yield of the solvent fraction was obtained from the chloroform fraction (40.75%) of the leaf extract of *Cordia africana*, followed by that of the aqueous fraction (37.2%), and the remaining (10.58%) was the *n*-butanol fraction.

### 3.2. Acute Oral Toxicity Test

In the acute toxicity study, the 80% methanol leaf extract of *C. africana* was found to be safe at the tested dose level of 2000 mg/kg as there was no death occurred within the first 24 hour as well as during the two weeks of follow-up period. Gross physical and behavioral observations of the experimental mice also revealed no visible signs of overt toxicity such as diarrhea, convulsions, lacrimation, hair erection, salivation, weight loss, lethargy, and paralysis.

### 3.3. Effect of Crude Extract of the Plant on the 4-Day Suppression Test

Results of the four-day suppression test showed that the leaf extract of *C. africana* had a significant antimalarial activity in *P. berghei*-infected mice. Analysis of the test results exhibited that percent suppression was 18.41, 44.16, and 51.19% at 200, 400, and 600 mg/kg dose levels of the extract, respectively. The highest suppression of the parasite was observed at 600 mg/kg dose of the extract. In addition, all dose levels of the crude extract indicated that there is a significant difference (*p* < 0.001) in chemosuppression effect as compared to doses of the extract as well as with the negative control (Table 1).

Besides, all the three doses of the extract showed statistically significant (*p* < 0.001) reduction of parasitemia compared to the negative control. However, the effect observed was much lesser than that of the positive control group. The standard drug, active chloroquine, cleared the parasite to undetectable level on day 4. The doses of the extract showed a significant difference (*p* < 0.001) in parasitemia reduction when compared to each other (Table 1).

With regard to mean survival time, the middle (400 mg/kg) and higher (600 mg/kg) dose of the plant extract were capable of significantly (*p* < 0.001) prolonging survival time of infected mice when compared to the negative control. While compared among the extract-treated groups, the higher dose (*p* < 0.001) showed a significant increase in survival time relative to the lower dose (200 mg/kg). The chloroquine-treated group showed a longer (*p* < 0.001) mean survival time when compared to vehicle- and extract-treated groups (Table 1).

The effect of the extract on body weight of *P. berghei*-infected mice is presented in Table 2. Analysis of percent change in body weight before and after the treatment revealed that the crude extract significantly reduced body weight loss at 400 mg/kg and 600 mg/kg (*p* < 0.001) as compared to the negative control group. In addition, comparison among the extract-treated groups indicated that mice treated with the intermediate and higher doses of the extract showed significant (*p* < 0.001) reduction in body weight relative to the lower dose on early malaria infection (Table 2).

**Figure 3:**
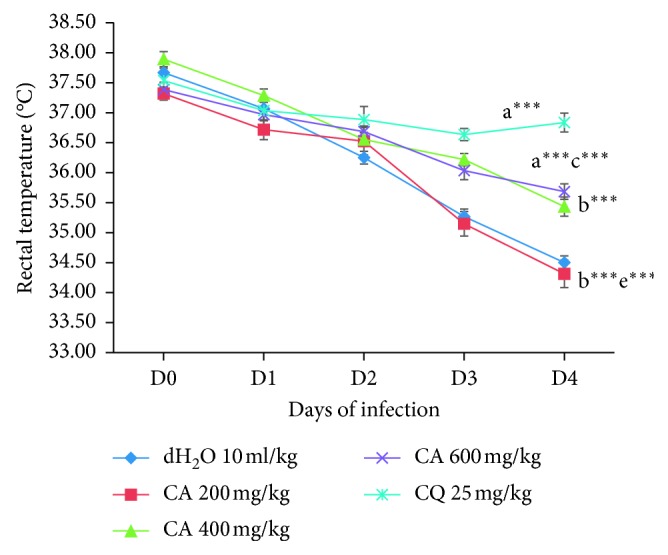
Effect of the 80% methanol crude leaf extract of *C. africana* on rectal temperature of *P. berghei*-infected mice on four-day suppression test. Data are expressed as mean ± SEM; *n* = 6; a, compared to 10 ml/kg dH_2_O; b, compared to CQ 25 mg/kg (positive control); c, compared to 200 mg/kg of the extract; d, compared to 400 mg/kg of the extract; e, compared to 600 mg/kg of the extract; ^*∗*^*p* < 0.05, ^*∗∗*^*p* < 0.01, ^*∗∗∗*^*p* < 0.001; CQ, chloroquine; CA, 80% methanol crude leaf extract of *Cordia africana*; dH_2_O, distilled water.

As depicted in Figure 3, all dose levels of the crude extract and chloroquine did not protect the reduction in body temperature following their respective treatment. The higher dose of the extract was the only dose-displaying statistically comparable effect (*p* < 0.001) in averting body temperature reduction as compared to the positive group and it also showed significant (*p* < 0.01) effect as compared to the lower dose group. However, the extract had not significantly prevented the reduction in body temperature in the lower (200 mg/kg) and medium (400 mg/kg) doses of the extract as compared to the negative control.

### 3.4. Effect of the Plant Crude Extract on Curative Test

The findings of the curative test regarding to percent parasite suppression were 32.56, 45.86, and 57.14% at dose levels of 200, 400, and 600 mg/kg, respectively (Table 3). Analysis of the percent suppression showed that the extract-treated groups had a significant suppression effect (*p* < 0.001) when compared to the negative control group. However, the positive control group was more effective (*p* < 0.001) than all the test doses of the extract. In addition, comparison between the three doses of the extract revealed a significant (*p* < 0.001) difference in percent parasite suppression.

The plant extract demonstrated antimalarial activity at all test doses, and the level of parasitaemia decreased with dose. Despite there was a progressive increment of mean parasitemia level during the course of treatment in all doses of the extract and the negative control group, the parasitemia level of the negative control group was relatively greater than that of the extract-treated group. On the contrary, after second dose administration, there was a gradual decrement of mean parasitemia level across days of treatment in the positive control group. On day 6, chloroquine completely eradicated the parasite to untraceable level (Table 3).

In addition, analysis of mean parasitemia level on day 7 indicated that the higher dose (600 mg/kg) of the extract-treated group showed significant (*p* < 0.001) difference as compared to the lower dose (200 mg/kg) of the extract-treated group, whereas the medium dose (400 mg/kg) of the crude extract had not shown statistically significant difference in parasitemia level as shown in Table 3.

The result of mean survival time indicated that the medium (400 mg/kg) and higher (600 mg/kg) doses of the extract prolonged their survival time of the respective groups significantly (*p* < 0.001) as compared to the negative control group. In addition, statistically significant (*p* < 0.001) prolonged survival time was also observed for the higher dose relative to the lower dose of the plant extract. Besides, the chloroquine-treated group exhibited a highly significant (*p* < 0.001) prolongation of survival time compared to all doses of the extract and vehicle-treated groups (Table 3).

With regard to body weight parameter, a reduction in body weight was exhibited at all the three dose levels of the plant crude extract as indicated in Table 4. Analysis of percent change in body weight, between pretreatment and posttreatment days, showed that all doses of the extract had a significant (*p* < 0.001) prevention of the reduction in body weight when compared to the negative control group. The higher (600 mg/kg) dose of the extract was the only dose that showed comparable effect in attenuating the reduction in body weight compared to the positive control, and it also revealed significant (*p* < 0.05) effect as compared to the lower (200 mg/kg) dose-treated group.

**Figure 4:**
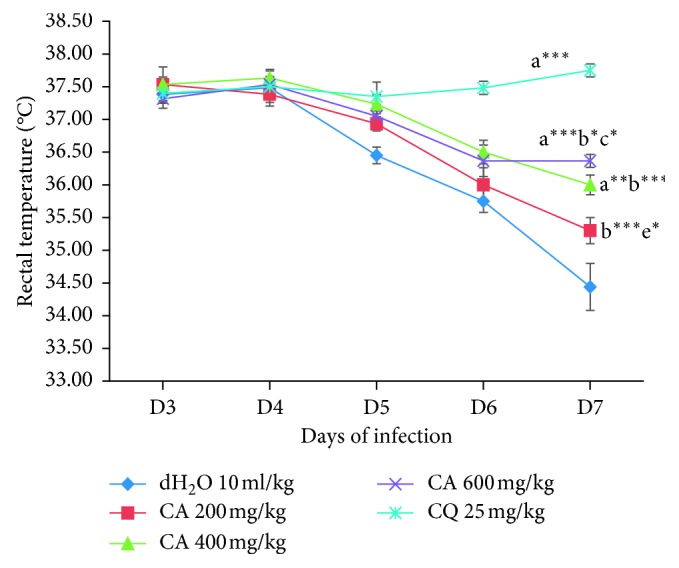
Effect of the 80% methanol crude leaf extract of *C. africana* on rectal temperature of *P. berghei*-infected mice in Rane's test. Data are expressed as mean ± SEM; *n* = 6; a, compared to 10 ml/kg dH_2_O; b, compared to CQ 25 mg/kg (positive control); c, compared to 200 mg/kg of the extract; d, compared to 400 mg/kg of the extract; e, compared to 600 mg/kg of the extract; ^*∗*^*p* < 0.05; ^*∗∗*^*p* < 0.01; ^*∗∗∗*^*p* < 0.001; CQ, chloroquine; CA, 80% methanol crude leaf extract of *Cordia africana*; dH_2_O = distilled water.

As it is shown in Figure 4, none of the doses of the extract of *C. africana* significantly protected the reduction in body temperature as compared to the positive control. Analysis of percent change in body temperature, between days 3 and 7, indicated that the higher (*p* < 0.001) and middle (*p* < 0.01) doses of the extract caused a significant attenuation of decline in body temperature relative to the negative control group. Among the doses of the extract, only the higher dose of the extract revealed a significant (*p* < 0.05) prevention of decline in body temperature relative to the lower dose.

### 3.5. Effect of the Plant Crude Extract on Chemoprophylactic Test

The chemoprophylactic test result revealed that the crude leaf extract of *C. africana* had a considerable antimalarial activity in *P. berghei*-infected Swiss albino mice (Table 5). All evaluated dose levels showed statistically significant (*p* < 0.001) difference in percent parasite suppression as compared to the negative control group after prophylactic test. The parasite load suppression of the extract was found to be 23.22%, 41.02%, and 46.48% in mice pretreated with 200 mg/kg, 400 mg/kg, and 600 mg/kg, respectively. The highest percent suppression (98.03% (*p* < 0.001)) of parasitemia was noted by the standard drug relative to all the three extract doses despite complete eradication of the parasite was not achieved.

In addition, analysis of mean parasitemia level on day 3 showed that all dose levels of the extract had significant (*p* < 0.001) difference from that of the negative control. Among doses of the extract, both the higher (600 mg/kg) and middle (400 mg/kg) doses had significant (*p* < 0.001) difference in parasitemia level when compared to the lower dose of the extract (Table 5).

Furthermore, those mice pretreated with the higher and middle doses of the extract showed statistically significant (*p* < 0.001) difference in prolonging the survival time from that of the negative control. At the three dose levels evaluated, those mice pretreated with the higher (600 mg/kg, *p* < 0.001) and middle (400 mg/kg, *p* < 0.05) doses of the extract showed a significant prolongation of survival time when compared to the lower (200 mg/kg) dose of the extract (Table 5).

As indicated in Table 6, all the test extract-treated groups as well as both the vehicle- and chloroquine-treated groups showed decline in body temperature following their respective treatment. Analysis of percent body temperature change, between day 0 and 3, indicated that both the standard drug and the higher dose (600 mg/kg) of the extract significantly (*p* < 0.001) prevented rectal temperature reduction compared to the negative control group.

With respect to body weight, the middle (400 mg/kg) and higher (600 mg/kg) doses of the crude extract averted body weight loss significantly (*p* < 0.001) in comparison with the negative control group. In addition, the chloroquine treated group had a significant effect as compared to the lower dose (*p* < 0.001) extract and vehicle (*p* < 0.001) treated groups. Like in the four-day suppression test, statistical significant difference was not found among doses of the extract. Only the higher dose of the extract had a comparable effect of the chloroquine on attenuating body weight reduction with no statistical significant difference (Table 6).

### 3.6. Effect of Solvent Fractions on the 4-Day Suppression Test

All doses of chloroform, *n*-butanol, and aqueous fractions significantly suppressed the parasitemia level (*p* < 0.001) compared to the negative control group after evaluated in the four-day suppression test. The parasite suppression effect of the fractions was increased in a dose-dependent manner as shown in Table 7. The higher percent chemosuppression observed with the *n*-butanol fraction (55.62%), followed by the chloroform fraction (45.04%) and aqueous fraction (28.56%). However, the effect was still significantly lower (*p* < 0.001) relative to the chloroquine. The mice treated with chloroquine were completely free from the parasite on day 4.

The mean survival time of the *n*-butanol fraction revealed significant effect (*p* < 0.001) when compared to the negative control group, but the effect was higher (*p* < 0.001) at 400 mg/kg dose of the fraction. However, only the middle (200 mg/kg) and lower (100 mg/kg) doses of the *n*-butanol fraction showed statistically significant (*p* < 0.001) difference in prolonging survival time of the treated mice as compared to the negative control group. In addition, the higher (400 mg/kg, *p* < 0.01) and middle (200 mg/kg, *p* < 0.05) doses of the aqueous fraction showed a significant effect compared to those of the vehicle-treated group. Unlike the standard drug-treated group, none of the mice treated with the fractions were cured from the infection (Table 7).

With regard to body weight parameter, none of the doses of the fractions of *Cordia africana* significantly prevented body weight reduction of the parasite-infected mice as compared to the chloroquine-treated group. The body weight loss increased with increasing the doses of the fractions in early malaria infection model as indicated in Table 8. Analysis of percent body weight change revealed that all dose levels of the *n*-butanol fraction had a significant (*p* < 0.001) attenuation of reduction in body weight compared to that of the vehicle-treated group. Both the middle (200 mg/kg) and higher (400 mg/kg) test doses of the chloroform fraction displayed significant effect in comparison (*p* < 0.001) with the negative control group, whilst no apparent difference was observed between the test doses of the aqueous fraction and the vehicle-treated mice. However, chloroquine had improved body weight significantly (*p* < 0.001) when compared to all doses of the three fractions (Table 8).

**Figure 5:**
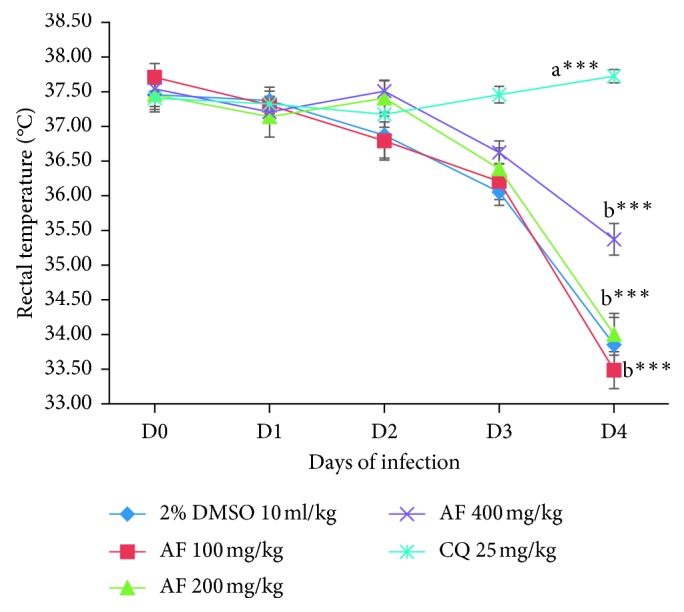
The effect of aqueous fraction of *C. africana* leaf extract on rectal temperature of *P. berghei*-infected mice on four-day suppression test. Data are expressed as mean ± SEM; *n* = 6; a, compared to 10 ml/kg 2% DMSO; b, compared to CQ 25 mg/kg (positive control); c, compared to 100 mg/kg of the fraction; d, compared to 200 mg/kg of the fraction; e, compared to 400 mg/kg of the fraction; ^*∗*^*p* < 0.05; ^*∗∗*^*p* < 0.01; ^*∗∗∗*^*p* < 0.001; AF, aqueous fraction; CQ, chloroquine; 2% DMSO, 2% dimethyl sulfoxide.

**Figure 6:**
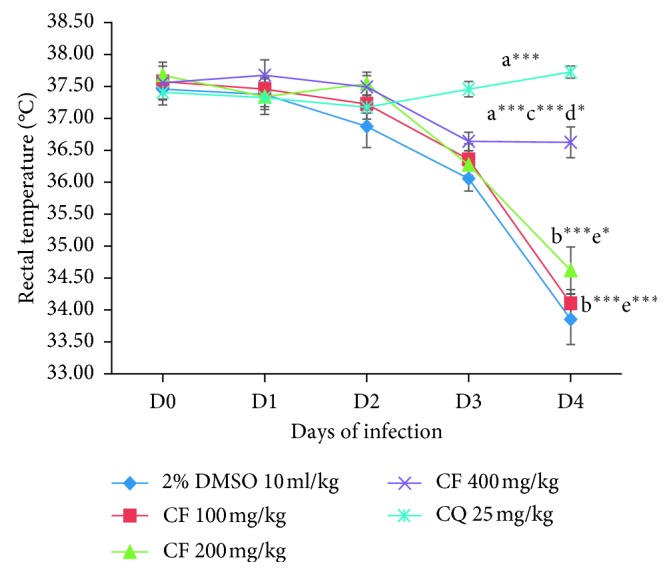
The effect of chloroform fraction of *C. africana* leaf extract on rectal temperature of *P. berghei*-infected mice on four-day suppression test. Data are expressed as mean ± SEM; *n* = 6; a, compared to 10 ml/kg 2% DMSO; b, compared to CQ 25 mg/kg (positive control); c, compared to 100 mg/kg of the fraction; d, compared to 200 mg/kg of the fraction; e, compared to 400 mg/kg of the fraction; ^*∗*^*p* < 0.05; ^*∗∗*^*p* < 0.01; ^*∗∗∗*^*p* < 0.001; CF, chloroform fraction; CQ, chloroquine; DMSO, dimethyl sulfoxide.

**Figure 7:**
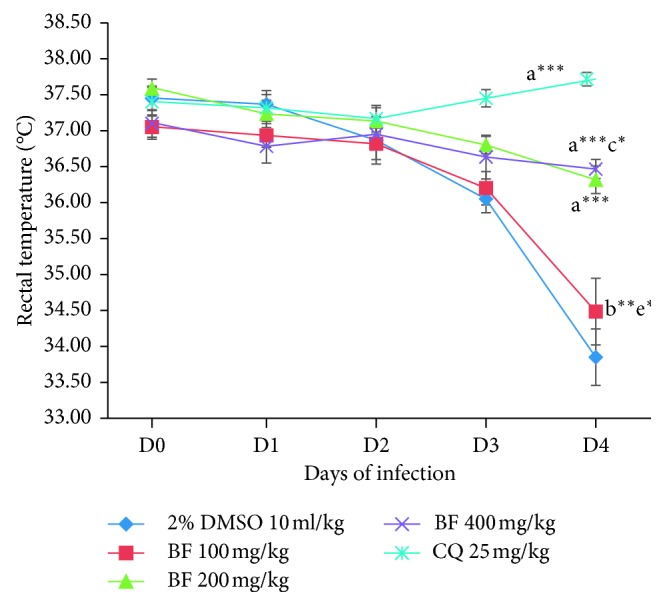
The effect of *n*-butanol fraction of *C. africana* leaf extract on rectal temperature of *P. berghei*-infected mice on four-day suppression test. Data are expressed as mean ± SEM; *n* = 6; a, compared to 10 ml/kg 2% DMSO; b, compared to CQ 25 mg/kg (positive control); c, compared to 100 mg/kg of the fraction; d, compared to 200 mg/kg of the fraction; e, compared to 400 mg/kg of the fraction; ^*∗*^*p* < 0.05; ^*∗∗*^*p* < 0.01; ^*∗∗∗*^*p* < 0.001; BF, *n*-butanol fraction; DMSO, dimethyl sulfoxide.

All the three fractions did not significantly protect the drop in body temperature as compared to the positive control group as depicted in Figures 5–7. Analysis of percent body temperature change indicated all dose levels of the *n*-butanol fraction had statistical significant (*p* < 0.001) difference in prevention of decrement in body temperature compared to the vehicle-treated group. However, both the chloroform and aqueous fractions were not able to prevent the reduction in body temperature as compared to the negative control group. While the positive control group had a significant (*p* < 0.001) difference in maintaining the normal body temperature as compared to the negative control as well as to all the test doses of the three fractions.

## 4. Discussion

Medicinal plants, which are practically unlimited sources of novel pharmacologically active compounds, have incredible role with many therapeutic applications. As a result, about half of all drugs in clinical use today are plant based [36].

The acute toxicity result of the present study indicated that the lethal dose of the plant extract could be higher than 2000 mg/kg in mice as per the OECD guideline number 425 [28]. This suggests that acute oral administration of the extract is safe. The result was also in consistent with the previous toxicity study [23] done on the root bark of the plant.

In all the three malaria models, determination of percent suppression of parasitemia is the most reliable parameter [24]. An antimalarial test compound is considered as an active compound (possesses antimalarial activity) when it shows a parasitaemia suppression of greater than or equal to 30% in standard screening studies [37].

The four-day suppression test is a standard test commonly used for *in vivo* antimalarial activity screening [38]. It is the most widely used test, in which the efficacy of a compound is assessed by comparison of parasitemia load and mean survival time between untreated and treated mice [39]. Besides, the test using *P. berghei*-infected mice provides a better prediction of antimalarial efficacy of drugs for human use in many studies of antimalarial drugs [31].

With the four-day suppression test, the crude extract significantly inhibited the level of parasitemia at 400 mg/kg and 600 mg/kg dose levels as compared to the negative control group. This finding confirms that the potential schizontocidal activity of the plant extract in early malaria infection. The parasite suppression exhibited by the extract was comparable with studies done on the 80% methanol seed extarct of *Brassica nigra* (L.) Koch. (Brassicaceae) [40] and the root extract of *Indigofera spicata* [41]. Similar study on *C. trichotoma*, belongs to the same species of *C. africana*, reported that the extract had significant parasitaemia suppression [42]. Many species of the genus *Cordia* such as *Ehretia acuminata* R.Br, *Cynoglossum zeylanicum* Thunb. Ex. Lehm, and *Cordia myxa* also reported to have antimalarial activity in different *in vitro* studies [43, 44].

Previous phytochemical investigations revealed that the study plant possesses compounds such as flavonoids, saponins, phenols, terpenoids, sterols, cumarins, tannins, and triterpenes that might be responsible for its antimalarial activity [10, 45]. Moreover, gas chromatography mass spectrometery (GC-MS) analysis of ethyl acetate fractions of the leaves of *C. africana* showed the presence of different compounds like methyl ester; 2-hydroxy-4-methylbenzaldhyde; neophytadiene; pentadecanoic acid; 1,2-benzene dicarboxilic acid; ester; and octadecanoic acid [46]. Those compounds may also contribute for the antimalarial effects of the study plant.

Saponins, flavonoids, cumarins, tannins, and phenols of the study plant have been shown to be bioactive and possess desirable pharmacologic activities such as antioxidant, antinociceptive, and anti-inflammatory activities [14–16, 47]. Antioxidant properties of the plant extract were analyzed by measuring its total flavonoids and total phenols [10, 17, 48], which may also contribute to the antimalarial activity. Antioxidant activity can inhibit heme polymerization, as heme has to be oxidized before polymerization and then unpolymerised heme is very toxic for the parasite [49].

According to the findings by Isan et al., *C. africana* leaf and stem bark possess radical-scavenging effect due to the 15-lipoxygenase inhibitory activity (inflammation mediator) and some nonbiological free radicals such as 1,1-diphenyl-2-picrylhydrazyl radicals and the ferric ion-reducing antioxidant power [17, 47]. The antimalarial activity of *C. africana* may be due to inhibition of the release of the inflammatory mediators associated with malaria such as cytokines [50, 51]. Furthermore, *C. africana* traditionally used for its “*michi*” [52] and fever reduction ability [9]. Those observations further strengthen the notion that the study plant has antimalarial activity.

In the curative test, the *C. africana* extract demonstrated significant reduction in parasitemia level at all test doses as compared to the vehicle-treatment group. The extract had shown a significant percentage of suppression with maximum of 57.14% at the dose of 600 mg/kg, indicating the curative potential of the extract. Even though the extract failed to completely eradicate the parasite, it caused a progressive reduction in parasitaemia level starting from the third dose (day 5) as compared to the negative control. The results are in agreement with the previous study done on the 80% methanol extract of leaves of *Balanites rotundifolia* (Van Tiegh.) Blatter (Balanitaceae) [53].

Prophylactic test of the extract had shown antimalarial activity with a relatively lower percentage of parasitemia suppression when compared to the four-day suppression and curative tests. Even though the middle (400 mg/kg) and higher (600 mg/kg) doses of the extract demonstrated significant (*p* < 0.001) chemosuppression effect on the level of parasitemia compared to the negative control, the positive control group caused (98.03%) parasite suppression when compared to the negative control. The parasite chemosuppression effect of the extract demonstrated in the present malaria prophylactic study was in line with previous studies on the 80% methanol leaf extract of *Calpurnia aurea* (Fabaceae) [35], *Justicia schimperiana* Hochst. Ex Nees (Acanthaceae) [30], and *Syzygium guineense* (Myrtaceae) [54].

Chloroquine completely eradicated the parasite in both the four-day suppression and Rane's tests, which is similar to the effect of the standard drug obtained in other studies using *Croton macrostachyus* Hochst. (Euphorbiaceae) [24] and *Ajuga remota* (Lamiaceae) [55]. Despite the chemosuppression exhibited by the standard drug in this test was relatively lowest when compared to its activities in early and established infection. This low chemosuppression activity of the standard drug could probably be resulted from rapid metabolisms that inactivate the active component of the drug or this could be attributed to the short half-life of chloroquine in rodents compared to human [56].

The fractions further showed that varying degrees of antimalarial activity after the four-day suppression test. The *n*-butanol fraction was found to be the most active fraction among the fractions. The chloroform fraction was found to have the second highest antimalarial activities from the fractions. However, the aqueous fraction showed the lowest percentage of suppression in the test. This indicates the difference in the type and concentration of the bioactive secondary metabolites in the fractions, suggesting the possible localization of active ingredients in the *n*-butanol and chloroform fractions. This effect might have also been associated with the existence of saponins, phenols, flavonoids, and terpenoids in the two fractions [26].

Furthermore, high levels of percent chemosuppression were produced at high doses of the chloroform and *n*-butanol fractions. This indicates the presence of concentrated secondary metabolites in higher doses of the two fractions. In contrary, the aqueous fraction produced the lowest inhibition of parasitemia level in the four-day suppression test. This could probably emanate from the absence of most of the secondary metabolites from this fraction that appeared to be responsible for the observed antimalarial activity. The results are similar to the former studies in which the butanol fraction had high activity than the chloroform and aqueous fractions [27, 34]. Furthermore, the least and highest parasitemia suppression effects of the aqueous and *n*-butanol fractions, respectively, are similar to the previous studies on the solvent fractions of the methanolic root extract of *Dodonaea angustifolia* (Sapindaceae) [26].

The mean survival time is important to evaluate the antimalarial activity of plant extracts [31]. The longest mean survival time of the mice was strongly associated with the maximum parasitemia inhibition, and this parameter was used to evaluate the efficacy of the antimalarial plant extract [57]. In all the three evaluated models, the middle (400 mg/kg) and higher (600 mg/kg) doses of the crude extract showed statistically significant (*p* < 0.001) difference in prolonging survival time as compared to the negative control group.

Moreover, the mean survival time of mice treated with all the fractions of the plant material was longer compared to the respective negative controls. However, only the middle (200 mg/kg) and higher (400 mg/kg) doses of the *n*-butanol and chloroform fractions showed significantly (*p* < 0.001) longer survival time than the negative control. Prolonging the mean survival time of the study mice indicates that the extracts/fractions suppressed *P. berghei* and reduced the overall pathologic effect of the parasite on the study mice [58]. However, neither the extracts nor the fractions cured the infection. This could be due to recrudescence of *P. berghei* parasites after apparent cure related to short-lived actions by the extracts/fractions [34]. This finding is comparable with results of other studies [40, 41].

In addition to the above parameters, there are also other parameters used when evaluating antimalarial activity of a given plant extract in the animal model. Ideal plant extracts with antimalarial activity are expected to prevent the reduction of body weight and temperature due to the rise in parasitemia [39].

Body weight loss is the one feature of rodent malaria infections. All dose levels of the crude extracts and solvent fractions did not prevent loss in body weight in *P. berghei*-infected mice as compared to the positive control group. However, comparison analysis of percent body weight change, between days 0 and 4, indicated that the crude extract significantly lessened body weight loss at the middle and higher dose levels of the crude extracts and solvent fractions as compared to the negative control group. Weight decrement has been associated with disturbed metabolic function and hypoglycemia associated with malaria [58]. According to the reported findings in [24], the loss in body weight indicates that the plant could have appetite suppression effect. This appetite suppression activity might be ascribed to saponins, flavonoids, glycosides, and phenolic compounds found in the crude extracts and solvent fractions, which support the present study. This result is comparable with the results of other studies [30, 35].

Body temperature reduction is another parameter used to assess the feature of rodent malaria infection. In the present study, all the crude extracts and fractions had been failed to prevent the reduction of parasite-induced rectal temperature compared to the reference drug. This could be attributed to the effect of the extract as it may have hypothermic effect on the treated mice. However, both the extracts and fractions at higher dose levels protected the mice from body temperature reduction which is significantly compared to the negative control in all the tests models. However, the extracts and fractions had not significantly prevented the reduction in body temperature in the lower and the middle doses of the extract from that of the negative control. The effects on rectal temperature in this study are comparable to those of previously reported findings by [34, 35, 59].

## 5. Conclusion

The results of the present study revealed that the 80% methanol leaf extract of *C. africana* has a significant antimalarial activity in a dose-dependent manner in all the three evaluated animal models. It further indicated that the solvent fractions of the crude extracts possessed varying degree of antimalarial activity after the four-day suppression test. The *n*-butanol fraction was found to be the most active fraction in the tested model. Acute toxicity test of the plant extract also exhibited that the lethal dose is above 2 g/kg.

## Figures and Tables

**Table 1:** Effect of crude extracts on parasitemia level, suppression, and mean survival time in the four-day suppression test.

Group	Doses (mg/kg)	% parasitaemia (mean ± SEM)	% suppression	Mean survival time (days)
dH_2_O	10 ml/kg	45.97 ± 0.56	0.00	6.33 ± 0.33
CQ	25	0 ± 0.00^a3^	100^a3^	30.00 ± 0.00^a3^
CA	200	37.51 ± 0.39^a3,b3,d3,e3^	18.41^a3,b3,d3,e3^	7.17 ± 0.31^b3,e3^
CA	400	25.67 ± 0.48^a3,b3,c3^	44.16^a3,b3,c3,e3^	8.50 ± 0.22^a3,b3^
CA	600	22.44 ± 1.16^a3,b3,c3^	51.19^a3,b3,c3,d3^	9.67 ± 0.42^a3,b3,c3^

Data are expressed as mean ± SEM; *n* = 6; a, compared to 10 ml/kg dH_2_O (negative control); b, compared to CQ 25 mg/kg; c, compared to 200 mg/kg of the extract; d, compared to 400 mg/kg of the extract; e, compared to 600 mg/kg of the extract; ^1^*p* <  0.05; ^2^*p* < 0.01; ^3^*p* < 0.001; CQ, chloroquine; CA, 80% methanol crude leaf extract of *Cordia africana*; dH_2_O, distilled water. Numbers refer to dose in mg/kg/day.

**Table 2:** Effect of crude extracts on body weight in the four-day suppression test.

Group	Doses (mg/kg)	Body weight (gram)		% change
		D0	D4	
dH_2_O	10 ml/kg	34.17 ± 0.85	32.45 ± 1.00	−5.03
CQ	25	33.96 ± 1.13	34.34 ± 1.14	1.12^a3^
CA	200	34.17 ± 0.69	32.50 ± 0.73	−4.89^b3,d3,e3^
CA	400	33.55 ± 0.59	32.78 ± 0.55	−2.31^a3,b3,c3^
CA	600	34.08 ± 0.52	33.60 ± 0.51	−1.39^a3,b2,c3^

Data are expressed as mean ± SEM; *n* = 6; a, compared to 10 ml/kg dH_2_O (negative control); b, compared to CQ 25 mg/kg (positive control); c, compared to 200 mg/kg of the extract; d, compared to 400 mg/kg of the extract; e, compared to 600 mg/kg of the extract; ^1^*p* < 0.05; ^2^*p* < 0.01; ^3^*p* < 0.001; CQ, chloroquine; CA, 80% methanolic crude leaf extract of *Cordia africana*; D0, pretreatment value on day 0; D4, posttreatment value on day 4; dH_2_O, distilled water. Numbers refer to dose in mg/kg/day.

**Table 3:** % of parasitemia, suppression, and survival time of crude extracts in Rane's test.

Group	Doses (mg/kg)	D3	D4	D5	D6	D7	% suppression	Mean survival time (day)
dH2O	10 ml/kg	10.39 ± 0.95	23.06 ± 2.58	27.93 ± 2.59	35.13 ± 0.46	44.37 ± 1.75	0.00	6.67 ± 0.21
CQ	25	11.67 ± 0.65	7.09 ± 0.49	1.82 ± 0.29	0.00 ± 0.00	0.00 ± 0.00^a3^	100.00	30.00 ± 0.00^a3^
CA	200	9.42 ± 0.63	20.93 ± 1.62	22.58 ± 1.31	24.69 ± 0.78	29.93 ± 1.38^a3,b3,e3^	32.56	8.33 ± 0.33^b3,e3^
CA	400	12.08 ± 0.57	21.34 ± 0.88	20.42 ± 0.90	21.73 ± 0.50	24.02 ± 1.99^a3,b3^	45.86	9.83 ± 0.40^a3,b3^
CA	600	13.78 ± 0.78	18.29 ± 1.06	19.24 ± 1.94	18.72 ± 1.11	19.02 ± 0.48^a3,b3,c3^	57.14	10.50 ± 0.43^a3,b3,c3^

Data are expressed as mean ± SEM; *n* = 6; a, compared to 10 ml/kg dH_2_O (negative control); b, compared to CQ 25 mg/kg (positive control); c, compared to 200 mg/kg of the extract; d, compared to 400 mg/kg of the extract; e, compared to 600 mg/kg of the extract; ^1^*p* < 0.05; ^2^*p* < 0.01; ^3^*p* < 0.001; CQ, chloroquine; CA, 80% methanol crude leaf extract of *Cordia africana*; dH_2_O, distilled water. Numbers refer to dose in mg/kg/day.

**Table 4:** Body weight of mice treated with crude extracts in Rane's test.

Group	Doses (mg/kg)	Body weight (gram)		% change
		D3	D7	
dH_2_O	10 ml/kg	28.28 ± 1.16	25.51 ± 0.93	−9.77
CQ	25	28.18 ± 0.77	28.54 ± 0.76	1.25^a3^
CA	200	33.13 ± 0.88	31.36 ± 0.93	−5.37^a3,b3,e1^
CA	400	33.16 ± 0.45	32.54 ± 0.43	−1.86^a3,b2^
CA	600	31.28 ± 1.21	30.94 ± 1.27	−1.09^a3,c1^

Data are expressed as mean ± SEM; *n* = 6; a, compared to 10 ml/kg dH_2_O (negative control); b, compared to CQ 25 mg/kg (positive control); c, compared to 200 mg/kg of the extract; d, compared to 400 mg/kg of the extract; e, compared to 600 mg/kg of the extract; ^1^*p* < 0.05; ^2^*p* < 0.01; ^3^*p* < 0.001; D3, pretreatment value on day 3 after infection; D7, posttreatment value on day 7; CQ, chloroquine; CA, 80% methanol crude leaf extract of *Cordia africana*; dH_2_O, distilled water. Numbers refer to dose in mg/kg/day.

**Table 5:** Effect of crude extracts on % parasitemia level, suppression, and survival time in prophylactic test.

Group	Doses (mg/kg)	% parasitaemia (M ± SEM)	% suppression	Mean survival time (days)
dH_2_O	10 ml/kg	43.87 ± 0.90	0.00	6.50 ± 0.22
CQ	25	0.86 ± 0.43^a3^	98.03	30 ± 0.00^a3^
CA	200	33.68 ± 0.71^a3,b3,d3,e3^	23.22	7.33 ± 0.33^b3,d1,e3^
CA	400	25.87 ± 0.40^a3,b3,c3^	41.02	8.83 ± 0.31^a3,b3,c1^
CA	600	23.48 ± 0.62^a3,b3,c3^	46.48	9.17 ± 0.31^a3,b3,c3^

Data are expressed as mean ± SEM; *n* = 6; a, compared to 10 ml/kg dH_2_O (negative control); b, compared to CQ 25 mg/kg (positive control); c, compared to 200 mg/kg of the extract; d, compared to 400 mg/kg of the extract; e, compared to 600 mg/kg of the extract; ^1^*p* < 0.05; ^2^*p* < 0.01; ^3^*p* < 0.001; CQ, chloroquine; CA, 80% methanol crude leaf extract of *Cordia africana*. Numbers refer to dose in mg/kg/day.

**Table 6:** Effect of crude extracts on rectal temperature and body weight of mice before and after inoculation in prophylactic test.

Group	Doses (mg/kg)	Weight (gram)		% change	Temperature (°C)		% change
		D0	D3		D0	D3	
dH_2_O	10 ml/kg	31.98 ± 1.09	29.37 ± 0.94	−8.16	36.62 ± 0.10	33.82 ± 0.34	−7.65
CQ	25	32.04 ± 1.14	31.75 ± 1.10	−0.60^a3^	37.12 ± 0.20	36.70 ± 0.07	−1.12^a3^
CA	200	31.57 ± 0.96	30.07 ± 0.96	−4.77^b2^	36.89 ± 0.13	35.25 ± 0.28	−4.45
CA	400	29.73 ± 0.99	28.87 ± 0.97	−3.43^a3^	37.23 ± 0.15	35.77 ± 0.16	−3.93
CA	600	30.79 ± 0.97	30.22 ± 1.00	−1.84^a3^	37.60 ± 0.18	36.85 ± 0.15	−1.99^a3^

Data are expressed as mean ± SEM; *n* = 6; a, compared to 10 ml/kg dH_2_O (negative control); b, compared to CQ 25 mg/kg (positive control); c, compared to 200 mg/kg of the extract; d, compared to 400 mg/kg of the extract; e, compared to 600 mg/kg of the extract; ^1^*p* < 0.05; ^2^*p* < 0.01; ^3^*p* < 0.001; CQ, chloroquine; CA, 80% methanol crude leaf extract of *Cordia africana*; D0, pretreatment value on day 0; D3, posttreatment value on day 3. Numbers refer to dose in mg/kg/.

**Table 7:** % of parasitemia, suppression, and survival time solvent fractions in four-day suppression test.

Group	Doses (mg/kg)	% parasitaemia (mean ± SEM)	% suppression	Mean survival time (days)
2% DMSO	10 ml/kg	52.69 ± 2.13	0.00	6.50 ± 0.22
CQ	25	0.00 ± 0.00^a3^	100	30.00 ± 0.00^a3^
AF	100	44.08 ± 1.72^a1,b3^	16.34	7.33 ± 0.33^b3^
AF	200	40.82 ± 0.80^a3,b3^	22.53	8.00 ± 0.26^a1,b3^
AF	400	37.64 ± 1.28^a3,b3^	28.56	8.17 ± 0.31^a2,b3^
BF	100	36.51 ± 2.04^a3,b3,d1,e3^	30.71	7.67 ± 0.21^b3,e3^
BF	200	27.28 ± 1.20^a3,b3,c1^	48.22	9.00 ± 0.37^a3,b3^
BF	400	23.39 ± 1.48^a3,b3,c3^	55.62	10.33 ± 0.42^a3,b3,c3^
CF	100	43.49 ± 0.85^b3,d2,e3^	17.45	7.50 ± 0.22^b3,e3^
CF	200	31.70 ± 1.95^a3,b3,c2^	39.84	8.33 ± 0.21^a3,b3^
CF	400	28.96 ± 2.49^a3,b3,c3^	45.04	9.67 ± 0.42^a3,b3,c3^

Data are expressed as mean ± SEM; *n* = 6; a, compared to 10 ml/kg 2% DMSO (negative control); b, compared to CQ 25 mg/kg (positive control); c, compared to 100 mg/kg of the fraction; d, compared to 200 mg/kg of the fraction; e, compared to 400 mg/kg of the fraction; ^1^*p* < 0.05; ^2^*p* < 0.01; ^3^*p* < 0.001; AF, aqueous fraction; BF, *n*-butanol fraction; CF, chloroform fraction; CQ, chloroquine; DMSO, dimethyl sulfoxide.

**Table 8:** Body weight of *P. berghei*-infected mice treated with solvent fractions in four-day suppression test.

Group	Doses (mg/kg)	Body weight (gram)		% change
		D0	D4	
2% DMSO	10 ml/kg	32.62 ± 1.46	29.25 ± 1.30	−10.32
CQ	25	26.73 ± 1.62	27.27 ± 1.53	2.02^a3^
AF	100	32.33 ± 1.02	29.21 ± 1.10	−9.67^b3^
AF	200	32.84 ± 2.09	30.70 ± 1.97	−6.53^b3^
AF	400	33.32 ± 1.45	31.62 ± 1.71	−5.10^b2^
BF	100	30.68 ± 0.89	29.88 ± 0.86	−2.62^a3,b1^
BF	200	26.73 ± 1.62	26.61 ± 1.67	−0.42^a3^
BF	400	34.20 ± 0.83	34.47 ± 0.76	0.79^a3^
CF	100	28.82 ± 0.97	26.68 ± 0.87	−7.43^b3,e1^
CF	200	30.04 ± 1.05	29.02 ± 1.02	−3.38^a3,b1^
CF	400	32.20 ± 1.00	31.50 ± 1.05	−2.16^a3,c1^

Data are expressed as mean ± SEM; *n* = 6; a, compared to 10 ml/kg 2% DMSO (negative control); b, compared to CQ 25 mg/kg (positive control); c, compared to 100 mg/kg of the fraction; d, compared to 200 mg/kg of the fraction; e, compared to 400 mg/kg of the fraction; ^1^*p* < 0.05; ^2^*p* < 0.01; ^3^*p* < 0.001; AF, aqueous fraction; BF, *n*-butanol fraction; CF, chloroform fraction; CQ, chloroquine; D0, pretreatment value on day 0; D4, posttreatment value on day 4; DMSO, dimethyl sulfoxide.

## Data Availability

The datasets generated during and/or analyzed during the current study are available from the corresponding author on reasonable request.

## Abbreviations

ANOVA: Analysis of variance
*C. africana*: *Cordia africana*
dH_2_O: Distilled water
DMSO: Dimethyl sulfoxide
OECD: Organization for Economic Cooperation and Development
MST: Mean survival time
SPSS: Statistical Package for the Social Sciences.
